# Prevalence of Symptomatic Knee Osteoarthritis in Saudi Arabia and Associated Modifiable and Non-Modifiable Risk Factors: A Population-Based Cross-Sectional Study

**DOI:** 10.3390/healthcare11050728

**Published:** 2023-03-02

**Authors:** Omar W. Althomali, Junaid Amin, Tolgahan Acar, Syed Shahanawaz, Alanazi Talal Abdulrahman, Dalia Kamal Alnagar, Meshari Almeshari, Yasser Alzamil, Kamal Althomali, Noorah Alshoweir, Othman Althomali, Monira I. Aldhahi, Bodor H. Bin Sheeha

**Affiliations:** 1 Department of Physiotherapy, College of Applied Medical Sciences, University of Ha’il, Ha’il P.O. Box 2440, Saudi Arabia; 2 Department of Mathematics, University of Ha’il, Ha’il P.O. Box 2440, Saudi Arabia; 3 Department of Statistics, University of Tabuk, Tabuk 71491, Saudi Arabia; 4 Department of Diagnostic Radiology, College of Applied Medical Sciences, University of Ha’il, Ha’il P.O. Box 2440, Saudi Arabia; 5 Forensic Medicine, Saudi Ministry of Health, Taif P.O. Box 1829, Saudi Arabia; 6 Department of Physiotherapy, Alhada Armed Forces Hospital, Taif 26792, Saudi Arabia; 7 Department of Family Medicine Program, Saudi Ministry of Health, Taif P.O. Box 1829, Saudi Arabia; 8 Department of Rehabilitation Sciences, College of Health and Rehabilitation Sciences, Princess Nourah bint Abdulrahman University, P.O. Box 84428, Riyadh 11671, Saudi Arabia

**Keywords:** ageing, osteoarthritis, knee pain, prevalence, risk factors, gender differences, obesity

## Abstract

Objective: This study aimed to determine the prevalence of knee osteoarthritis (OA) in Saudi Arabia and the association between knee OA and modifiable and non-modifiable risk factors. Methods: A self-reported, population-based, cross-sectional survey between January 2021 and October 2021 was conducted. A large, population-representative sample (n = 2254) of adult subjects aged 18 years and over from all regions of Saudi Arabia was collected electronically using convenience sampling. The American College of Rheumatology (ACR) clinical criteria were used to diagnose OA of the knee. The knee injury and osteoarthritis outcome score (KOOS) was used to investigate the severity of knee OA. This study focused on modifiable risk factors (body mass index, education, employment status, marital status, smoking status, type of work, previous history of knee injury, and physical activity level) and non-modifiable risk factors (age, gender, family history of OA, and presence of flatfoot). Results: The overall prevalence of knee OA was 18.9% (n = 425), and women suffered more compared to their male counterparts (20.3% vs. 13.1%, *p* = 0.001). The logistic regression analysis model showed age (OR: 1.06 [95% CI: 1.05–1.07]; *p* < 0.01), sex (OR: 2.14 [95% CI: 1.48–3.11]; *p* < 0.01), previous injury (OR: 3.95 [95% CI: 2.81–5.56]; *p* < 0.01), and obesity (OR: 1.07 [95% CI: 1.04–1.09]; *p* < 0.01) to be associated with knee OA. Conclusions: A high prevalence of knee OA underlines the need for health promotion and prevention programmes that focus on modifiable risk factors to decrease the burden of the problem and the cost of treatment in Saudi Arabia.

## 1. Introduction

Osteoarthritis (OA) is the most common type of arthritis. It is a complex disorder that can affect the articular cartilage, bones, ligaments, and synovium. It contributes to degenerative and reparative processes and inflammation of the joint [1]. OA may affect different body joints, both proximal and distal (large, medium, and small joints), and most commonly occurs in the knee joint [2]. Several risk factors can increase the likelihood of having knee OA, and these can be divided into non-modifiable and modifiable factors [3]. There are six main well-known categories of modifiable risk factors: obesity and overweight, comorbidities (diabetic, depression, and cardiovascular disease), occupational factors, physical activity, biomechanical factors, and dietary exposure. Treatment should target the modifiable risk factors, as it is possible to reduce pain disability [3].

According to the International Classification of Functioning, Disability, and Health (ICF) framework, knee OA leads to activity limitation and participation restriction as well as impairment [4]. It is considered the primary cause of physical disability in the general population [5,6]. Physical disability resulting from pain and lack of functional capability decreases quality of life and raises the risk for more morbidity. Global statistics show that around 250 million people worldwide are affected by knee OA. In Saudi Arabia, it is one of the most common and growing health situations [7]. It is necessary to consider the prevalence of OA to understand the impact of the disease on society. Recent studies in Saudi Arabia have shown that knee OA increases with age, reaching up to 60.6% in people aged 66–75 years compared with 30.8% in those aged 46–55 years [8]. Other studies have shown that 39.75% of the population, including 53.3% of males and 60.9% of females, suffers from knee OA [1,9].

Prior studies related to the prevalence of OA were conducted in Saudi Arabia with some limitations. Those studies introduced threats to internal validity, and the data collected were from local regions or cities with a small sample size that could not represent the general population of the kingdom [1,8,10]. Moreover, gender-based differences were not addressed, and diagnostic criteria for OA were mainly based on radiological findings. Interestingly, a previous study showed 50% of subjects to be clinically asymptomatic with a radiographic finding and vice versa [11]. In Saudi Arabia, there is still insufficient data taken from a large sample size on knee OA and its risk factors. The clinical guidelines also do not recommend routine X-rays to diagnose OA [12]. Therefore, the current study uses clinical criteria to diagnose knee OA and includes participants in all regions of the kingdom so that there is an optimal number of participants to ensure a population-representative sample. Furthermore, the prevalence of OA in general has variations based on race and ethnicity [13]. Therefore, it is imperative to obtain an updated prevalence of knee OA and identify the modifiable risk factors for timely prevention and early intervention. The findings of the current study will also help health agencies and stakeholders to plan educational and preventive programmes to address the modifiable risk factors to ease the social-economic burden of OA.

The present study aims to determine the prevalence of symptomatic knee OA in Saudi Arabia and examine the association of knee OA with modifiable and non-modifiable risk factors. The secondary objective is to compare affected with non-affected individuals with knee OA using knee injury and osteoarthritis outcome score (KOOS).

## 2. Methods

### 2.1. Study Design and Participants

This population-based cross-sectional study was conducted between January and October 2021 among the Saudi general population. The study was approved by the research ethics committee of the University of Hail (Ethical approval no: H-2021-009). A convenience sampling method was used to collect data from all 13 regions of Saudi Arabia. Written informed consent was obtained from each participant before participation. Adult individuals aged 18 years and above were included. Individuals who had severe mental disorders and physical disabilities or deformities in the lower limbs were excluded. Individuals with severe mental disorders were excluded due to their inability to give informed consent and the desired information. Individuals with physical disabilities or deformities were also excluded due to the potential for their existing disability or deformity to affect pain, stiffness, and loss of function. The required sample size was calculated based on the previously published equation N = Z^2^P(1 − P)/d^2^, with a confidence interval of 95% [14]. Z (confidence level) value of 95% was selected since this is the most commonly used [14]. P (prevalence) was considered to be 0.22 (22%) based on two previous studies, one of which showed 16% of knee OA prevalence on a global scale [15], and the second investigated the prevalence of OA among Gulf Cooperation Council countries (average of studies for knee OA equal to 27%) [16]. This led us to take the average of the two studies (21.5%), which was rounded to 22%. The d (precision) was considered to be 0.018, being more conservative [14], and this led to 2035 participants, and around 11% (219 participants) were added to avoid any missing data.

### 2.2. American College of Rheumatology (ACR) Knee OA Assessment Criteria

OA is a pathological condition affecting the structures of the entire joint, such as cartilage degeneration, bone remodelling, osteophyte production and synovial inflammation that cause pain, stiffness, oedema and loss of function [17]. To diagnose knee OA, ACR clinical criteria were used. The criteria defined knee OA as pain felt in the greatest number of days over the previous 30 days accompanied by three of the following: (1) age of 51 years and above, (2) bony enlargement, (3) 30 min of joint stiffness, (4) bony tenderness and (5) crepitus [18].

### 2.3. Implementation of the Assessment Criteria

To address the current study’s aim and apply the ACR clinical assessment criteria, a self-reported survey was conducted, and a closed-ended questionnaire was designed. The questionnaire consisted of three sections. The first section contained demographic characteristics, lifestyle, and health-related issues. Collected information included gender (male or female), age (years), weight (kg), height (m), education (illiterate, primary, intermediate, high school, diploma, bachelor’s degree, or higher degrees), work type (office work, fieldwork, both office and fieldwork, retired, housewife, or unemployed), marital status (single or married), smoking habits (yes or no), previous knee injuries (yes or no), presence of flat feet (yes, no, or I do not know), family history of OA (yes, no, or I do not know), and physical activity level (inactive, low intensity, moderate intensity, or high intensity).

In the second section, questions related to ACR clinical criteria were asked [18]. The questions were as follows: (1) “Have you felt pain in one or both knees in most of the previous 30 days?” (2) “In which knee do you have pain (right, left, both, or no pain)?” (3) “How long have you had the pain?” (4) “Do you feel pain when pressing or compressing your knee/knees?” (5) “Do you think your knee/knee bones is/are larger than normal (enlarged)?” (6) “Does/do your knee/knees produce the sound of clicking or crepitus?” (7) “Do you think your knee/knees feel stiff for the first 30 min in the morning?” The third section included the KOOS scale. All questions were addressed in Arabic since KOOS has been shown to be valid and reliable in the Arabic language [19].

### 2.4. KOOS Scale

KOOS was used to collect and investigate the severity of the knee OA and to compare, according to the American College of Rheumatology (ACR) clinical criteria, individuals with knee OA with non-OA individuals and individuals with knee pain without knee OA diagnosis. The psychometric properties of the KOOS scale have been assessed, and it has been found to be a reliable and valid instrument for assessing knee and associated problems [20].

The subscales of KOOS are pain (5 items), symptoms (4 items), ADL (9 items), sport/recreation (3 items), and QOL (2 items). The total KOOS is based on the individual score calculated for each subscale. Each item is scored ranging from 0 to 4 (0 = none, 1 = mild, 2 = moderate, 3 = severe, and 4 = extreme). The maximum score is 100, indicating no problem, while 0 indicates extreme problems. An Excel spreadsheet downloaded from the official website (http://www.koos.nu/index.html, accessed on 1 December 2020) was used to calculate KOOS.

### 2.5. Scoring to Diagnose Individuals with Knee OA

**Figure 1:**
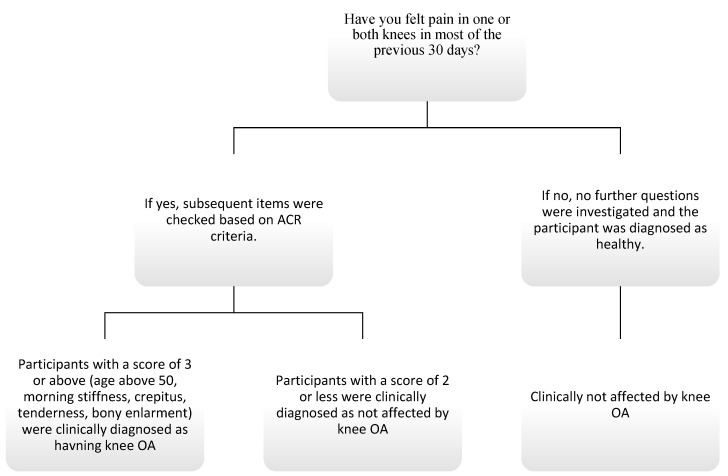
Flow diagram of knee OA diagnosis based on ACR criteria.

The first step was to identify individuals who had suffered knee pain in the majority of the previous 30 days and answered “yes” to the question “Have you felt pain in one or both knees in most of the previous 30 days?”, for which they were given a score of 1. In the second step, a score of 1 was given for the presence of any of the following symptoms: crepitus (“Do you feel pain when pressing or compressing your knee/knees?”), bony enlargement (“Do you think your knee/knee bones is/are larger than normal?”), bony tenderness (“Do you feel pain when pressing or compressing your knee/knees?”), the presence of 30 min of morning joint stiffness (“Do you think your knee/knees feel stiff for the first 30 min in the morning”), and age above 50 years (“How old are you?”). If the total score reached 3 or above, the ACR was fulfilled, and the participant was diagnosed as having clinical knee OA. If the question in the first step concerning the presence of knee pain in the majority of the previous 30 days had been answered as no or yes, and the total score was less than 3, the participant was diagnosed as healthy (Figure 1).

### 2.6. Data Collection

The electronic data collection was executed via an online Google form. The link to the form was shared with potential participants through their WhatsApp, Twitter, and email accounts. The link was republished more than once to increase the response rate. The link was shared with individuals in all regions of Saudi Arabia (Makkah Region, Riyadh Region, Eastern Region, Asir region, Jazan Region, Medina Region, Al-Qassim Region, Tabuk Region, Ha’il Region, Najran Region, Al-Jawf Region, Al-Bahah Region, and Northern Borders Region).

### 2.7. Statistical Analysis

The collected data were extracted from the Google form into a Microsoft Excel (version 16.33) spreadsheet and then exported to SPSS version 25 (SPSS Inc. Chicago, IL, USA). Body mass index (BMI) was divided into four categories based on the WHO classification (underweight < 18.5, normal = 18.5–24.9, overweight = 25–29.9, and obese > 29.9) [21]. Individuals were grouped by age into three categories (18–30, 31–49, and ≥50) to enable comparison. Descriptive analysis was performed for categorical data and presented as frequencies and percentages. The prevalence of knee OA was compared between the different demographics, lifestyles, and health-related characteristics using the chi-square test. KOOS subscales and total KOOS were compared for individuals with no incidence of knee OA to individuals with knee OA, and a comparison was conducted between individuals with knee pain and no OA and individuals with knee OA using an independent sample *t*-test after checking the for normality. Cohen’s d was calculated to show the effect size and interpreted as large (≥0.8), medium (0.5–0.79), and small (0.2–0.49). Forward binominal logistic regression was used to investigate the risk factors related to knee OA that were significant when the chi-square test was applied. Age and BMI were entered into the model as continuous variables. A *p*-value less than 0.05 was set as a statistically significant level.

## 3. Results

A total of 2254 individuals from the 13 regions of Saudi Arabia responded to the questionnaire. The age of respondents ranged from 18 to 80 years (mean 35 ± 13.11). Most of the respondents (80.88%) were females, and 44.23% were aged 18–30 years; 60.74% were married, and 35.58% were of a normal body weight. Most of the participants (60.03%) had completed a bachelor’s level of education, 29.41% were office workers, and 27.06% were unemployed (Table 1). The majority of respondents (89.66%) reported no previous injury to the knee (ACL, meniscus). Family history of knee OA was reported in 63.75% of the participants, and 6.83% reported having flat feet (Table 2).

A total of 1262 (55.99%) participants reported having knee pain. Approximately 21.21% had had knee pain for 1–5 years, while 3.06% reported having it for longer than 15 years; 2.62% had been absent from work for more than 15 days in the previous 12 months due to knee pain. The prevalence of knee OA based on ACR clinical criteria was 18.86% (n = 425) (Table 2).

The prevalence of knee OA significantly differed between gender, age group, marital status, BMI category, previous knee injury, family history of OA, presence of flat feet, educational level, smoking habits, and physical activity level (Table 3). The prevalence of knee OA increased with age; 6.82% of participants aged 18–30 years were affected by knee OA, whereas 45.77% of the participants 50 years and above were affected. The prevalence of knee OA was significantly higher among females than among their male counterparts (20.25% vs. 12.99%, *p* < 0.01). The prevalence of knee OA was higher in married (25.57%, *p* < 0.01) and obese (34.30%) individuals. Conversely, the prevalence of knee OA was not common in smokers compared with non-smokers (19.42% vs. 11.11%, *p* = 0.012). Moreover, the prevalence was highest in illiterate individuals (84.62%), and the least was observed in individuals with master’s/Ph.D. degrees (14.74%, *p* < 0.01). The prevalence of knee OA was 49.57% (*p* < 0.01) among those with previous injuries to the knee and 31.82% (*p* < 0.01) and 22.30% (*p* < 0.01) in respondents who reported having flat feet and family history of OA, respectively.

The logistic regression model was statistically significant (*p* < 0.01). The model showed age, gender, previous injury, level of physical activity, education level, smoking, family history of OA, and BMI to be associated with increased or decreased prevalence of knee OA. Females had more than twofold the risk for developing knee OA than males (OR: 2.14 [95% CI: 1.48–3.11]; *p* < 0.01). Ageing was also associated with an increase in the risk for knee OA (OR: 1.06 [95% CI:1.05–1.07]; *p* < 0.01). Previous injury to the knee (ACL, meniscus) was also found to be a risk factor for knee OA (OR: 3.95 [95% CI: 2.81–5.56]; *p* < 0.01). Smoking was found to decrease the risk for knee OA (OR: 0.51 [95% CI: 0.27–0.96]; *p* < 0.01). Individuals with higher degrees were found to have less risk for knee OA in comparison to those who were illiterate. Higher BMI was found to be associated with an increase in the risk for knee OA (OR: 1.07 [95% CI: 1.04–1.09]; *p* < 0.01). Interestingly, individuals with moderate levels of physical activity were found have decreased risk for knee OA compared with inactive individuals, while those with a high level of physical activity showed an increased level of risk compared with inactive individuals. Individuals with a family history of OA showed a higher risk for developing knee OA than individuals with no family history of OA (Table 4).

Interestingly, when comparing non-OA participants with those with knee OA using KOOS, all subscales showed significant differences with *p*-values less than 0.01 and a large effect size. On comparing non-OA individuals with knee pain and individuals with OA, there was a significant difference in all KOOS subscales and total KOOS scores with a large effect size (Table 5).

## 4. Discussion

Knee OA is a common, progressive, and degenerative disease that affects the daily lives of many people across the globe. Pain, stiffness, and limited mobility associated with the condition have negative influences on people’s quality of life [22]. Therefore, the overarching aim of this study is to determine the prevalence of symptomatic knee OA and the associated risk factors in Saudi Arabia.

Our study reported a high prevalence of knee OA (18.86%, n = 425). A recent systematic review and meta-analysis summarizing 88 previous studies showed similar findings [16], highlighting that the pooled global prevalence of knee OA for those aged 15 and above was 16%, ranging from 14.3% to 17.8% [15], highlighting that the pooled global prevalence of knee OA for those aged 15 and above was 16%, ranging from 14.3% to 17.8%. Our study also showed that the prevalence increases with shifting the minimum age. A previous study in Al-Qaseem city reported the prevalence of knee OA using the same diagnostic criteria as were used in our study and showed a prevalence of 13% [8], which was 4.9% lower than the findings in this study.

Importantly, the current study showed a statistically significant association between OA and age, gender, BMI, previous knee injury, level of education, level of physical activity, family history of OA, and smoking. Previous studies have also shown age [23], female sex [24], obesity [25], genetic factors [26], and previous injury [27] to be linked with the increased risk for knee OA. Interestingly, the current study found smoking to be protective and that it can reduce the risk for OA. A previous systematic review was also aligned with the current study’s findings [28]. Moreover, higher education levels were protective and reduced the risk for developing knee OA, which is also in agreement with our study [29]. On comparing the risk factors in the current study, the results showed that previous history of knee injury ranked as the highest risk by a 3.95 odds ratio.

Our evidence suggests that age increases in the risk for osteoarthritis. The age of the subjects in our study ranged from 18 to 80 years, and the occurrence of knee OA increased up to 45.77% among persons aged over 50 years compared with 6.82% in those aged between 18 and 30 years. This can potentially be attributed to ageing cellular and physiological change that is associated with decreased muscle strength and mass, poor proprioception, and cartilage thinning [30]. It has been reported by the WHO Scientific Group on Rheumatic Diseases that an estimated 10% of the world’s population aged 60 years and older have significant clinical problems that can be attributed to OA [31].

Our study found a high prevalence of knee OA in females and obese individuals. Previous studies have also shown an increasing trend for knee OA in females, which is likely to be due to hormonal factors and anatomical and kinematic differences in females [32]. Obesity is responsible for additional overloading of the weight-bearing joints, which further contributes to triggering the wear-and-tear process inside joints among individuals with high BMI [33]. A recent study in Saudi Arabia showed similar results regarding the increased risk for developing knee OA in individuals with higher BMI [34]. A high prevalence of obesity has been reported in Saudi females (33.5%) compared with males (24.1%) [35]. Based on these statistics, Saudi females with obesity may be more likely to have additional risk for knee OA development. Moreover, advancing age in Saudi females with obesity may further pose a risk for knee OA. Hence, modifiable risk factors should be targeted to reduce the risk for developing knee OA in the future and lower the burden of OA in the Saudi community. A previous study showed that weight loss resulted in a reduction in the risk for developing OA and symptoms in individuals affected by OA [36]. A weight-loss programme can be used as a proactive strategy to prevent and manage many non-communicable diseases, including OA, in Saudi Arabia.

Several factors can explain the reduced risk for individuals with a higher education level and smokers developing OA. Smoking may promote the proliferation of chondrocytes, improve the expression of cartilage-specific type 2 collagen, and have anti-inflammatory effects [28]. A previous study could not conclude a reason for education level reducing the risk for developing OA [29]. However, higher education could mean more knowledge about disease prevention, while those with lower education most likely work in office jobs, which may require standing for long periods and bending. These claims are just speculatory in nature and need to be proved. Notably, the prevalence of knee OA in individuals with flatfoot was significantly higher (31.82%) than in those with normal feet (17.03%), supporting reported evidence that bilateral flat feet are significantly associated with worse OA-related knee pain and disability [37]. This may be explained by changing the load distribution on the knee when the foot is flat. A previous study showing knee pain and knee OA cartilage damage to be associated with flatfoot supports our earlier claim [38].

Despite the current study using clinical criteria to diagnose individuals with knee OA, the KOOS subscales showed that there was a significant difference between those affected with knee OA and those who were healthy and those who were healthy with knee pain, which strengthens the study. The current study showed that individuals with knee OA had 58.24 ± 18.32, 55.29 ± 16.28, 58.74 ± 20.15, 38.61 ± 25.46, and 46.75 ± 22.05 scores for pain, symptoms, ADL, sport, and QOL, respectively. A previous study that investigated the reliability of KOOS in the Saudi population with knee OA showed similar results for pain (45.6 ± 18.6), symptoms (52.9 ± 21.3), ADL (47.4 ± 20.1), sport (17.7 ± 18.9), and QOL (31.3 ± 16.8) subscales [39]. These findings are in line with a study that showed poor KOOS outcomes with knee OA after a joint injury compared to uninjured controls [40]. Interestingly, the association between the prevalence of knee OA and level of physical activity showed that moderate levels reduced the risk for knee OA compared to sedentary lifestyle, whereas high levels led to increased risk for knee OA. According to the Physical Activity Guidelines for Americans, moderate levels of physical activity (150 min/week of moderate-intensity exercise in bouts lasting ≥10 min) and lower levels of physical activity (at least 45 total minutes/week of moderate-intensity exercise) were associated with improved function and gait speed in OA patients. The reverse impact of high levels of physical activity on knee OA may be explained by the nature of the physical activity, where high-impact activity and a large number of weight-bearing exercises may lead to joint destruction [41].

### Strengths and Limitations

This study has certain strengths, one of which is that it is the first population-representative study from Saudi Arabia with a large sample size. This study also informs the fundamental knowledge and highlights the prevalence of knee OA in the Saudi population. Another strength is its use of valid clinical criteria for detecting knee OA. Nevertheless, measuring the association between both modifiable and non-modifiable risk factors would have further strengthened this study. Using an online form to collect the data has the advantages of reducing cost and being able to reach remote areas, although it raises concerns about the accuracy and reliability of the data. The modifiable risk factors associated with knee OA will further guide the design of effective interventions to reduce the burden of disease in the community.

However, some limitations should be acknowledged, such as the current study’s use of a cross-sectional design, which has a major limitation in reporting causal explanations. The current study also used a convenience sampling technique, which may reduce the representativeness of the sample in the general population. Furthermore, due to a lack of logistic support, only a self-reported method was used, which may have generated reporting bias in the study, especially since an exaggerated difference in prevalence between sexes has been reported in the literature, as females may be more likely to report OA [42]. Future research with clinical-based diagnoses by specialized health providers is warranted to attain robust findings. Likewise, future studies should explore other risk variables that may increase the risk for developing knee OA, such as the type of shoes worn or knee adduction moment during activity. A previous study showed that higher knee adduction movement led to progression in the knee OA by increasing the load on the medial side of the knee [43] by increasing the load on the medial side of the knee. Using footwear such as lateral wedge insoles can reduce knee adduction movement [44].

## 5. Conclusions

The study reveals a high prevalence of knee OA among the Saudi population. It contributes to a better understanding of the modifiable and non-modifiable risk factors associated with symptomatic knee OA. The study identifies associated non-modifiable risk factors (age, gender, and family history of OA) and modifiable risk factors (BMI, previous knee injury, smoking, physical activity level, and level of education) with knee OA. The information from this study is helpful for identifying people at risk for developing knee OA and targeting them by designing prevention plans such as weight-loss strategies and improving their physical activity levels. The findings of the current study can also help clinicians, policymakers, and stakeholders to target the associated modifiable risk factors explored in this study to decrease the burden and treatment cost of knee OA. The design of the current study (cross-sectional and using an online survey) may limit its generalisability, and therefore, longitudinal studies are needed.

## Figures and Tables

**Table 1:** Characteristics of the respondents (n = 2254) included in the study.

Characteristic	Number (%)
Gender	
Male	431 (19.12)
Female	1823 (80.88)
Marital status	
Married	1369 (60.74)
Single	885 (39.26)
BMI (kg/m^2^)	
Underweight	118 (5.24)
Normal	802 (35.58)
Overweight	646 (28.66)
Obese	688 (30.52)
Education	
Illiterate	13 (0.58)
Primary	26 (1.15)
Intermediate	60 (2.66)
High school	422 (18.72)
Diploma	224 (9.94)
Bachelor’s degree	1353 (60.03)
Master’s/Ph.D. (higher degree)	156 (6.92)
Type of work	
Unemployed	610 (27.06)
Office work	663 (29.41)
Fieldwork	464 (20.59)
Mixed (office and field)	112 (4.97)
Retired	97 (4.30)
Housewife	308 (13.66)
Smoking	
Smoker	2101 (93.21)
Non-smoker	153 (6.79)
Level of physical activity	
Inactive	1098 (48.71)
Low intensity (e.g., walking), requiring low effort	925 (41.04)
Moderate intensity (e.g., jogging), requiring moderate effort	149 (6.61)
High intensity (e.g., running), requiring high effort	82 (3.64)
Age (years)	
18–30	997 (44.23)
31–49	855 (37.93)
≥50	402 (17.83)

**Table 2:** Prevalence of OA and knee pain and associated risk factors.

Variable	Number (%)
Diagnosis of knee OA according to our study criteria	
No	1829 (81.14)
Yes	425 (18.86)
Duration of knee pain (years)	
No pain	992 (44.01)
<1	510 (22.63)
1–5	478 (21.21)
6–10	149 (6.61)
11–15	56 (2.48)
>15	69 (3.06)
Affected knee with pain	
No	992 (44.01)
Right side	432 (19.17)
Left side	358 (15.88)
Bilateral	472 (20.94)
Previous knee injury	
No	2020 (89.62)
Yes	234 (10.38)
Flat feet	
No	1773 (78.66)
Yes	154 (6.83)
I do not know	327 (14.51)
Sick leave in previous 12 months due to knee pain (days)	
No pain	992 (44.01)
No sick leave	1016 (45.08)
1–5	145 (6.43)
6–10	35 (1.55)
11–15	7 (0.31)
>15	59 (2.62)
Family history of OA	
No	463 (20.54)
Yes	1437 (63.75)
I do not know	354 (15.71)

**Table 3:** The prevalence and association of knee OA with different characteristics.

Variables	Non-OA	OA	*p*-Value *
	n (%)	n (%)	
Gender			<0.01
Male	375 (87.01)	56 (12.99)	
Female	1454 (79.75)	369 (20.25)	
Age (years)			<0.01
18–30	929 (93.18)	68 (6.82)	
31–49	682 (79.77)	173 (20.23)	
≥50	218 (54.23)	184 (45.77)	
BMI			<0.01
Underweight	112 (94.92)	6 (5.08)	
Normal	722 (90.02)	80 (9.98)	
Overweight	543 (84.06)	103 (15.94)	
Obese	452 (65.70)	236 (34.30)	
Previous knee injury (ACL, meniscus)			<0.01
No	1711 (84.70)	309 (15.30)	
Yes	118 (50.43)	116 (49.57)	
Family history of OA			<0.01
No	410 (88.60)	53 (11.40)	
Yes	1117 (77.70)	320 (22.30)	
I do not know	302 (85.30)	52 (14.70)	
Smoking			0.012
Non-smoker	1693 (80.58)	408 (19.42)	
Smoker	136 (88.89)	17 (11.11)	
Marital status			
Married	1019 (74.43)	350 (25.57)	<0.01
Single	810 (91.53)	75 (8.47)	
Flat feet			
No	1471 (82.97)	302 (17.03)	<0.01
Yes	105 (68.2)	49 (31.82)	
I do not know	253 (77.37)	74 (22.63)	
Level of physical activity			
Inactive	882 (80.33)	216 (19.67)	<0.01
Low intensity	738 (79.78)	187 (20.22)	
Moderate intensity	141 (94.63)	8 (5.37)	
High intensity	68 (82.93)	14 (17.07)	
Education			<0.01
Illiterate	2 (15.38)	11(84.62)	
Primary	10 (38.46)	16 (61.54)	
Intermediate	39 (65.00)	21 (35.00)	
High school	343 (81.28)	79 (18.72)	
Diploma	176 (78.57)	48 (21.43)	
Bachelor’s degree	1126 (83.22)	227 (16.78)	
Master’s/Ph.D. (higher degree)	133 (85.26)	23 (14.74)	

* *p*-value obtained from chi-square test.

**Table 4:** Binomial logistic regression results for knee OA with the different study characteristics.

Variable	B	S.E.	*p*-Value	OR	95% C.I.	
					Lower	Upper
Gender (female)	0.76	0.19	<0.01	2.14	1.48	3.11
Age	0.06	0.01	<0.01	1.06	1.05	1.07
BMI	0.07	0.01	<0.01	1.07	1.04	1.09
Smoker (yes)	−0.67	0.32	0.04	0.51	0.27	0.96
Previous injury to knee (ACL, meniscus) (yes)	1.37	0.17	<0.01	3.95	2.81	5.56
Education level (illiterate)	Reference					
Primary	−0.57	0.91	0.53	0.57	0.10	3.36
Secondary	−1.21	0.87	0.17	0.30	0.05	1.65
High school	−1.37	0.82	0.10	0.25	0.05	1.27
Diploma	−1.68	0.83	0.04	0.19	0.04	0.94
Bachelor’s degree	−1.66	0.81	0.04	0.19	0.04	0.93
Master’s/Ph.D. (higher degree)	−2.11	0.85	0.01	0.12	0.02	0.64
Family history of OA (no)	Reference					
Yes	0.49	0.18	0.01	1.63	1.15	2.32
I do not know	0.32	0.24	0.18	1.37	0.86	2.20
Level of physical activity (no)	Reference					
Low intensity	−0.08	0.13	0.53	0.92	0.72	1.19
Moderate intensity	−1.15	0.40	0.00	0.32	0.14	0.70
High intensity	0.90	0.34	0.01	2.47	1.28	4.77
Constant	−5.11	0.94	0.00	0.01		

**Table 5:** Comparison between non-OA and knee OA participants and between non-OA with knee pain and knee OA participants based on KOOS score.

KOOS	Diagnosis	Number	Mean ± SD	*p*-Value between Non-OA and OA	Effect Size	*p*-Value between Non-OA with Knee Pain and OA	Effect Size
Pain	Non-OA *	1829	88.40 ± 14.55	<0.01		<0.01	
	Knee pain without OA **	837	79.99 ± 15.37		1.96		1.32
	Knee OA ***	425	58.24 ± 18.32				
Symptoms	Non-OA	1829	80.91 ± 13.56	<0.01		<0.01	
	Knee pain without OA	837	78.04 ± 13.32		1.81		1.58
	Knee OA	425	55.29 ± 16.28				
ADL	Non-OA	1829	89.58 ± 14.41	<0.01		<0.01	
	Knee pain without OA	838	83.48 ± 15.93		1.97		1.42
	Knee OA	425	58.74 ± 20.15				
Sports	Non-OA	1829	81.90 ± 22.73	<0.01		<0.01	
	Knee pain without OA	838	71.12 ± 25.01		1.86		1.29
	Knee OA	425	38.61 ± 25.46				
QOL	Non-OA	1829	81.90 ± 20.66	<0.01		<0.01	
	Knee pain without OA	838	70.14 ± 21.35		1.68		1.08
	Knee OA	425	46.75 ± 22.05				
Total	Non-OA	1829	84.54 ± 14.43	<0.01		<0.01	
	Knee pain without OA	837	76.55 ± 15.15		2.19		1.56
	Knee OA	425	51.53 ± 17.52				

* Non-OA, without pain; ** Knee pain without OA, not diagnosed with ACR and knee pain other than OA; *** Knee OA, diagnosed with knee OA using ACR.

## Data Availability

The datasets generated and/or analysed during the current study are available from the corresponding author upon reasonable request.
